# Supplementary material for: A maternal germline mutator phenotype in a family affected by heritable colorectal cancer
Source: Genetics. 2024 Oct 15;228(4):iyae166. doi: 10.1093/genetics/iyae166 (PMC11631438; doi:10.1093/genetics/iyae166)
Supplement: iyae166_Supplementary_Data [file iyae166_supplementary_data.zip › Supplementary_Methods_GENETICS-2024-307519.pdf]

Supplementary Methods: A maternal germline mutator phenotype in a family affected by heritable colorectal cancer

Candice L. Young[1,2*], Annabel C. Beichman[1*], David Mas-Ponte[1], Shelby L. Hemker[3], Luke Zhu[4], Jacob O. Kitzman[3], Brian H. Shirts[5], and Kelley Harris[1,6**]

[1]Department of Genome Sciences, University of Washington, 3720 15th Ave NE, Seattle, WA 98195

[2]Department of Molecular and Cellular Biology, University of Washington, 1705 NE Pacific St, Seattle, WA 98195

[3]Department of Human Genetics, University of Michigan, 1241 Catherine St, Ann Arbor, MI 48109

[4]Department of Bioengineering, University of Washington, 3720 15th Ave NE, Seattle, WA 98195

[5]Department of Laboratory Medicine and Pathology, University of Washington, 1959 NE Pacific St, Seattle, WA 98195

[6]Herbold Computational Biology Program, Fred Hutchinson Cancer Center, P.O. Box 19024, Seattle, WA 98109

[*]These authors contributed equally to this work

[**]Corresponding author (harriske@uw.edu)

Table of Contents

1. Genome sequencing and SNP calling.. 2
2. Using surrogate parents for DNM calling.. 3
3. Accessible Genome Size Estimation.. 4
4. Filtering... 5
5. IGV inspection... 6
6. Read-backed phasing... 7
7. Calculation of each family's expected mutation burden in the absence of a genetic mutator effect.. 9
8. Comparing our observed mutation counts to the null parental age model of Jónsson et al. (2017)... 11
9. Estimating the minimum mutator effect sizes that we have power to detect.................. 11
10. Mutational Signature Analysis... 13

1. Genome sequencing and SNP calling

All sequencing was conducted at the University of Washington Northwest Genomics Center (NWGC). Samples had a detailed sample manifest (i.e., identification number/code, sex, DNA concentration, barcode, extraction method). Initial quality control (QC) entailed DNA quantification, sex typing, and molecular "fingerprinting" using a 63-SNP OpenArray assay derived from a custom exome SNP set. This "fingerprint" was used to identify potential sample handling errors and provided a unique genetic ID for each sample, which eliminated the possibility of sample assignment errors. Samples failed if: (1) the total amount, concentration, or integrity of DNA was too low; (2) the fingerprint assay produced poor genotype data; or (3) sex-typing was inconsistent with the sample manifest. No samples failed quality control at this stage.

Library construction was automated in 96-well plate format. At least 750 ng of genomic DNA was subjected to a series of library construction steps utilizing the KAPA Hyper Prep kit (KR0961 v1.14). All library construction steps were automated on the Perkin Elmer Janus platform. Libraries were validated using the Biorad CFX384 Real-Time System and KAPA Library Quantification Kit (KK4824). Barcoded genome libraries are pooled using liquid handling robotics prior to loading. Massively parallel sequencing-by-synthesis with fluorescently labeled, reversibly terminating nucleotides was carried out on the NovaSeq sequencer. Variant calling was carried out by the NWGC. Their variant calling pipeline

combined a suite of Illumina software and other “industry standard” software packages (i.e., Genome Analysis ToolKit [GATK], Picard, BWA, SAMTools, and in-house custom scripts) and consisted of (1) alignment to human reference genome GRCh38DH using BWA-MEM (v0.7.15) (Li and Durbin 2009), (2) local realignment, (3) PCR duplicate removal (Picard MarkDuplicates; v2.6.0), (4) base quality score recalibration (BQSR) (GATK BaseRecalibrator; v3.7), (5) data merging, (6) variant detection, (7) genotyping, and (8) annotation.

The SeattleSeq Annotation Server (http://gvs.gs.washington.edu/ SeattleSeqAnnotation/), an automated pipeline, was used for annotation of variants derived from genome data. This publicly accessible server returned annotations including dbSNP rsID (or whether the coding variant was novel), gene names and accession numbers, predicted functional effect (e.g., splice-site, nonsynonymous, missense, etc.), protein positions and amino-acid changes, PolyPhen predictions, conservation scores (e.g., PhastCons, GERP), ancestral allele, dbSNP allele frequencies, and known clinical associations.

2. Using surrogate parents for DNM calling

We used GATK PossibleDeNovo as in the previous section on each informative “trio” of a child, a real parent if available, and one or two surrogate parents. For children whose parents’ genome sequences were both available (C11, C12, C31, and C32), we performed no surrogate DNM calling. For children whose mother’s genome was available but whose father’s genome was unavailable (C21, C22, C23, C41, C42), we called DNMs using the mother’s sequence plus each available relative as surrogate father. This resulted in two overlapping DNM call sets for each of the three siblings C21, C22, and C23, but just a single call set for C41 and C42. To generate each call set, we generated a positive mask file consisting of regions that we identified to be shared IBD between the child and the surrogate father, then called DNMs within the bounds of this positive mask minus the standard negative mask previously used to filter out low quality regions during standard DNM calling. We then merged together all call sets generated for the same child with different surrogate fathers.

To call mutations in each of the parents P1–P4, we ran PossibleDeNovo a total of nine times, each using a different combination of relatives as surrogate mother and father. Six of these runs involved a pair of two distinct relatives Pi and Pj, and the remaining three runs used the same sibling as both the surrogate mother and the surrogate father. For each run, a distinct positive mask was used to call mutations only in

regions where the child shared two distinct parental haplotypes with its pair of surrogate parents. In the case where the same relative was used as both surrogate mother and surrogate father, this meant regions where the child shared two distinct IBD tracts with the same surrogate parent, because the two relatives had inherited the same chromosome from both their mother and their father. As before, DNM calls from all nine runs were merged to generate the total call set for each individual.

We generated additional mutation calls from P1–P4 by using each sibling Pi as a "double surrogate parent" for each other sibling Pj. We performed double surrogate calling within regions where hap-IBD found that Pi and Pj shared two overlapping IBD tracts, which indicates that they inherited the same maternal chromosome and also the same paternal chromosome. Since GATK PossibleDeNovo is designed for use with two distinct parental genomes, we called candidate double-surrogate DNMs within these double-IBD regions by identifying sites where the child is heterozygous but the double surrogate parent is homozygous.

We filtered out all DNMs called at sites that appear in the accessible regions of multiple surrogate parent combinations but are not consistently called using all of those surrogate parent combinations. For example, if a putative DNM in C22's genome occurs at a locus that appears accessible for calling using either C21 or C23 as surrogate father, but that DNM is only called using C21 as surrogate father, it will be filtered out of the final call set.

3. Accessible Genome Size Estimation

Using both conventional Mendelian violation methods and our devised surrogate method, we derived the overall mutation rate for each offspring. Determining these rates required the computation of a denominator for each individual within the pedigree. This denominator represented the number of genomic sites where the read coverage was adequate (i.e., greater than 12 or less than 120) to ascertain a mutation, if present. Sites lacking confident inference of an individual's parental haplotype sequences were excluded.

For offspring without sequenced fathers, our focus shifted to chromosomal regions where the child had an identical paternal haplotype with at least one sibling. For example, in the offspring of P2 with three children, two children with adequate read coverage at a site were necessary to identify mutations at that

locus for both. For the parent generation, mutation identification depended on factors such as sufficient read coverage, successful haplotype reconstruction, and inheritance patterns. Using the surrogate method necessitated adjustments to the denominators based on the total length of shared parental haplotypes, leading to variable accessible base numbers for offspring in Families 2 and 4 and the parent generation (**Figure S3**).

4. Filtering

DNMs were subjected to a series of quality control steps to eliminate potential false positives (**Figure S1A**). Building on prior research findings (Bergeron et al. 2022), true germline DNMs are usually characterized by alternative allele read support, with a variant allele frequency (VAF) ranging from 30% to 70%, and lack reads from either parent. DNMs were only considered for further analysis if they adhered to these parameters:

- Displayed a read depth between 12 and 120 for all members of both full pedigree and surrogate pedigree trios.
- Were identified by GATK PossibleDeNovo as being present in the child but not in either parent.
- Exhibited a VAF of 30-70% in the child.
- Had no reads supporting the variant in either parent.
- Genotypes filtered with GATK recommended hard filters: QD > 2.0; FS < 60.0; MQRankSum > -12.5; ReadPosRankSum > -8.0;SOR < 3.0

DNMs located in centromeres, telomeres, and segmental duplications were further excluded. Only DNMs that appeared in unique, accessible regions of the genome were retained in the final dataset. Additionally, any DNM that overlapped with variants having a minor allele frequency (MAF) of 1% or higher in the 1000 Genomes Phase 3 dataset was excluded. For DNMs identified using surrogate parents, a sliding window methodology was employed to pinpoint sparse mutations. The stipulated criteria for this was a maximum of 7 mutations within a 15MB sliding window, advancing in increments of 3MB.

5. IGV inspection

In order to verify the mutation calls from both the full trio sequences and the resulting variants from families with surrogate parental sequences, we performed visual inspection of the resulting calls by inspecting the raw reads around the called de novo mutations.

We queried the original mapped sequences (bam files) to obtain all reads within 10kb (5kb slop) all pre-called de novo mutations in each trio of samples. When a mutation was detected in one of the families with a missing paternal genome we included all other samples in that trio that were used as a surrogate-paternal sequence, thus including multiple bam files as parental sequences.

The reduced files were then processed to filter low quality reads by selecting unduplicated sequences (-F 1024) and requiring a mapping quality higher than 20 (--min-MQ 20). To select informative reads used by GATK for variant calling, the unfiltered reads were also used to re-call variants using GATK HaplotypeCaller with the -bamout flag option that returns the informative reads for each call in bam format. The resulting variant files from this step were discarded and not used in any of the analysis. Note that if the algorithm would not return a mutation in that position there would be no informative reads available.

For each trio or surrogate-parent trio we generated a IGV report using igv-reports (github.com/igvteam/igv-reports) that outputs a HTML file containing small snippets of all called variants from the original vcf files. Each variant has 3 extra tracks per sample: (1) the original mapped sequence (bam file used in the mutation calling pipeline), (2) the filtered bams without duplicated or lower quality mapped reads, and (3) the bams of 'informative reads' yielded from the re-run of GATK HaplotypeCaller. These 3 tracks were included per sample in each trio, i.e. for a full trio a total of nine bam tracks will be included in the report while for a surrogate-parent trio the bams of all siblings and the available parents would be included. The reports included a 10Kb window around each variant and also included the allele count (AD, in each family) and the quality of the genotype (QD, in the original call).

Each variant in the IGV reports was then visually inspected to determine possible errors in the mutation dataset of each trio (**Figure S3**). The variants that failed our test were then classified according to their problematic features.

- Read evidence in the parental genomes, undetected due to indel realignment
- Read evidence in the parental genomes, undetected due to other reasons
- Unconventional or nuanced mapping
- Polymorphism evidence (as presence in dbSNP), for families with surrogate parents
- Polymorphism evidence (as presence in dbSNP), for families with surrogate parents
- A cluster of mutations (≥ 6 mutations per 50 bp), some or all of which have rsID annotations (indicative of misclassified germline mutations due to the surrogate-calling method)

This manual curation resulted in the number of DNMs being reduced by ~36% per individual.

6. Read-backed phasing

The tool Unfazed (v1.0.3) (Belyeu et al. 2021), a read-based phasing approach, was used to phase the de novo variants to maternal or paternal haplotypes. This approach required the existence of an "informative" inherited heterozygous variant that could be phased to a parent present on the same sequencing read as the DNM. This requirement resulted in 14-42% of DNMs being phased per individual (**Table S1**), a fraction typical for studies of phased de novo mutations.

Generation and analysis of simulated trio data for surrogate-method benchmarking

We randomly generated five "children" of these parents by generating recombination events using the recombination segments (S_i) from a chromosome map (M) which was downloaded from the Beagle (2021) resource page (see https://bochet.gcc.biostat.washington.edu/beagle/genetic_maps/). We compiled the code to simulate the recombination events and combine the datasets from the multisample VCFs here, www.github.com/davidmasp/meiosim. Specifically, we simulated the number of crossings (x) using a Poisson distribution with λ equal to the length of the segment, measured in centimorgans (Δc), multiplied by a recombination rate (R) of 0.01 (crossings/cM). We then obtained x crossings (K_i) from a uniform distribution covering the positions of the recombination segment:

$$M = \{S_i\}$$

$$S_i = (p_0, p_1), (c_0, c_1)$$

$$x_i = Pois(\lambda = \Delta c \cdot R)$$

$$K_i = unif_{\{p_o, p_1\}}(n = x_i)$$

An initial haplotype was chosen at random from each parent and the haplotype was then swapped at each recombination breakpoint. SNPs from the parental haplotypes were then propagated to the children.

We added DNMs to each simulated child by selecting a proband uniformly at random without replacement from Jónsson et al. (2017) and editing the simulated child's genome to include these DNMs.

We generated a short read BAM file consistent with each simulated genome using DWGSIM (0.1.15, www.github.com/nh13/DWGSIM), a tool that simulates sequencing reads from a reference genome and can incorporate custom germline variants. We defined an error rate of 0.001, a read length of 151 bp, and a target coverage of 30X. All other parameters were left as default. Other software used in this process were bcftools (1.19) and samtools (1.19).

We mapped and processed all simulated reads using SAREK (3.3.2) (Garcia et al. 2023). In brief, Sarek checks for quality and trims raw reads using fastqc (0.11.9) and fastp (0.23.4) (Chen et al. 2018); then maps with BWA-MEM1 (0.7.17-r1188) and further process them with Mark Duplicates and Base Quality Score Recalibration from the GATK suite (4.4.0.0) (McKenna et al. 2010). In addition to that, it measures the coverage using mosdepth (0.3.3) (Pedersen and Quinlan 2018) and integrates the quality reports with multiqc (1.15) (Ewels et al. 2016).

We then employed the same pipeline used for real families to process the jointly genotyped VCF file and simulated BAM files of the family. This involved applying the same filtering criteria for DNMs and utilizing identical genomic accessibility masks on the BAM files to calculate the final accessible genome denominators for each surrogate individual.

In simulating the surrogate method, we designated "sibling 0" as the child and performed DNM calling using all possible combinations of siblings 1-4 as surrogate parents, with scenarios including both the presence and absence of the real mother (HG00132) using GATK's PossibleDeNovo. We then generated IBD tracks using hap-IBD to create surrogate tracts for all sibling pairs, in order to identify all regions where siblings shared a maternal or paternal IBD tract with sibling 0.

To minimize the inclusion of likely false positives, we applied the density filter (as detailed in the "Filtering" section and depicted in **Figure S1B**) to retain only mutations in "sparse" genomic regions.

While we did not curate these mutation calls via IGV, we did apply two stringent filters to remove false positive calls: 1) excluding any DNMs called in sibling 0 if another sibling had the alternate allele at that site, and 2) excluding any DNMs not called in all surrogate parent combinations which had the appropriate IBD segments to make that region of the genome accessible. We additionally classified false positive calls by identifying regions where a mutation was called in one surrogate trio combination but not in another, despite being accessible for detection. This distinction helped to account for limitations in the surrogate method and differentiate between true de novo mutations and likely false positives.

To assess how the inclusion of additional siblings as surrogates affected the method's recall and precision, we conducted tests in two configurations:

- Group 1) one surrogate parent acting as the father with the real mother included, utilizing up to four siblings in various combinations (one sibling + mother, two siblings + mother, three siblings + mother, four siblings + mother)
- Group 2) two surrogate parents with no real parent, considering three potential combinations of sibling surrogates (two siblings used, three siblings used, four siblings used)

In each scenario, we subset the callset of true DNMs based on the accessible genome provided by these sibling combinations, thereby differentiating between false negatives and inaccessible mutations. We also applied a stepwise false positive filter by sites at which two or more siblings shared an alternate allele.

With each additional sibling included in the surrogate combinations, we assessed mutations that were accessible across multiple surrogate call sets but were called in fewer than expected, thus further reducing false positives. The mutation rate at each step was calculated similarly to the method described in the "Accessible Genome Size Estimation" section, with the number of true and false positives serving as the numerator and two times the size of the accessible genome as the denominator.

7. Calculation of each family's expected mutation burden in the absence of a genetic mutator effect

To correct for differences between Jónsson et al. (2017)'s accessible genome size (2.68×10^9 bp) and the accessible genome sizes of each individual in our study (which ranged from 1.28×10^9 bp to 2.67×10^9 bp), we multiplied each expected mutation count under the parental age model by $\frac{g_i}{g_J}$, the ratio of the accessible genome of individual i (g_i) to Jónsson et al. (2017)'s accessible genome size (g_J). When the accessible genome size of an individual is considerably smaller than that of Jónsson et al. (as is the case for the individuals whose DNMs were called using the surrogate method), this rescaling will reduce the count of each mutation type we expect to observe in the offspring (**Figure S6**).

In order to determine whether the families in Sherwood et al. (2023) are consistent with the model trained on the families sequenced by Jónsson et al. (2017), we repeated the above procedure for the families in that study. Sherwood et al. (2023) didn't report each individual's accessible genome size, but since they did not employ the surrogate-calling method, their accessible genome size should be comparable to that of Jónsson et al. (2017), and so we did not carry out accessible genome size rescaling for these individuals. For each individual sequenced in our study and the Sherwood et al. (2023) study, we computed the ratio of observed to expected mutation counts for each mutation type.

When carrying out comparisons based on the subset of mutations we were able to phase to maternal and paternal haplotypes, we further downscaled the expected mutation counts by the phasing success rate per individual, which ranged from 14-40% (**Table S1**).

The above calculations yielded estimates of the relative rate of each mutation type in families with pathogenic human *MUTYH* genotypes relative to control families. To compare these effect sizes to the effect sizes of murine *Mutyh* mutator alleles, we computed analogous observed-over-expected ratios using mice with different *Mutyh* genotypes previously analyzed by Sasani et al. (2022). To compute the average mutation rate of each mutation type c in mice with a mutagenic *Mutyh* genotype known as the "D" genotype, we added up mutations of type c from all mice with the "D" genotype and divided this count by the total number of generations these mice were inbred, which is the total number of generations over which they had the opportunity to accumulate mutations. In the same way, we estimated a relative rate of mutations of type c in mice with the "B" *Mutyh* haplotype. Finally, we estimated the rate of mutations of type c in a single strain known as BXD68 affected by a unique *Mutyh* hypermutator phenotype. For the "D" allele and the BXD68 hypermutator allele, we divided the relative rate of each mutation type by the "B" allele rate to estimate the effect size of each of these *Mutyh* variants on mutagenesis in the mouse germline.

8. Comparing our observed mutation counts to the null parental age model of Jónsson et al. (2017)

We used the Poisson cumulative distribution function (CDF) to determine whether the overall and per-mutation type DNM counts we observe are consistent with the parental age model, or whether we see significant elevations of any mutation type, particularly the C>A type associated with a defective MUTYH protein.

For each individual, we calculated $P(X \geq k \mid \lambda)$: the probability that a Poisson random variable X will generate a value greater than or equal to our observed mutation count k, given that it has mean λ equal to the expected count calculated based on the parental age model regressions from Jónsson et al. (2017) (as described above). We used *R*'s ppois() Poisson CDF function to calculate this probability. The ppois() function with the "lower.tail = F" flag gives the probability $P(X > k \mid \lambda)$, and we calculated that $P(X \geq k \mid \lambda) = P(X > k - 1 \mid \lambda)$, such that

$P(X \geq k \mid \lambda)$ = ppois(q = (ObservedMutationCount - 1), lambda = ExpectedMutationCount, lower.tail=F)

This approach was used to determine whether the total observed mutation counts per individual were significantly greater than what we'd expect under the null parental age model expectation. We separately carried out this analysis for each mutation type (C>A, C>G, C>T, A>G, A>T, A>C) per individual, per nuclear family, and for mutation counts phased to each parent (total counts and per-mutation type counts).

9. Estimating the minimum mutator effect sizes that we have power to detect

For each biallelic parent whose offspring might be affected by a C>A mutator phenotype, we calculated the minimum C>A mutator effect size that should be statistically detectable using the above one-tailed Poisson test (leading us to reject the parental age model from Jónsson et al. 2017). To calculate this

minimum effect size, we used the qpois() function in *R* to calculate the number of C>A mutations that should yield a p-value < 0.05, with λ estimated from the parental age model:

qpois(p = 0.05, λ = parental age model expected C>A count, lower.tail = F).

We then added +1 to the mutation count given by qpois() to calculate the number of mutations needed to be observed (x) such that $P(X \geq x \mid \lambda) < 0.05$. We call this number of mutations the "mutator detection threshold." We calculated separate thresholds for each child of a biallelic parent (including C11, C12, C21, C22, C23, C31, C32) and also calculated a cumulative threshold for detecting an elevated C>A mutation rate in each family with a biallelic parent (Families 1, 2 and 3). The detection threshold varies slightly across individuals and families based on parental age, the sex of the biallelic parent, and the childrens' total accessible genome size.

To estimate the minimum biallelic *MUTYH* allele effect size we should be powered to detect, we assigned all excess C>A mutations above the parental age model's expectations to the carrier parent:

$$\hat{x}_{C>A,\,CP} = x_{C>A} - E_{C>A,\,NCP}$$

where $x_{C>A}$ is the mutator detection threshold (minimum number of mutations for which $P(X \geq x \mid \lambda) <$ 0.05), $E_{C>A,\,NCP}$ is the C>A count expected for the non-carrier parent (NCP) under the parental age model, and $\hat{x}_{C>A,\,CP}$ is the contribution of C>A mutations from the carrier parent (CP) needed to reach the significance threshold x, assuming all excess C>A above the parental age model expectation are assigned to the carrier parent.

The minimum detectable effect size of the biallelic *MUTYH* genotype should then be

$$\frac{\hat{x}_{C>A,\,CP}}{E_{C>A,\,CP}},$$

where $E_{C>A,\,CP}$ is the expected number of C>A mutations contributed by the carrier parent under the parental age model.

We can also use this framework to estimate the effect size of the C>A mutator phenotype in the germline of each biallelic parent, again making the assumption that all excess C>A mutation counts above the parental age model expectation can be assigned to the carrier parent:

$$\widehat{O}_{C>A,\,CP} = O_{C>A,\,total} - E_{C>A,\,NCP}$$

where $O_{C>A,\,total}$ is the total observed C>A mutation count in an individual child or set of children of the same biallelic parent. As before, $E_{C>A,\,NCP}$ is the expected number of C>A mutations contributed by the non-carrier parent under the parental age model, and $\widehat{O}_{C>A,\,CP}$ is the estimate of how many C>A mutations are contributed by the carrier parent, assuming all excess C>A mutations are assigned to that parent.

The *MUTYH* effect size required to yield this number of mutations is then

$$\frac{\widehat{O}_{C>A,\,CP}}{E_{C>A,\,CP}}$$

where $E_{C>A,\,CP}$ is the expected number of C>A mutations contributed by the carrier parent under the parental age model.

10. Mutational Signature Analysis

Non-negative matrix (NMF) factorization was used to extract mutational signatures from the de novo 3-mer mutation spectra, either per-individual, or summed up per-family. *SigProfilerExtractorR* (v. 1.1.16), an R wrapper for *SigProfilerExtractor* (Islam et al. 2022), was used to carry out the analyses. The reference genome was set to "GRCh38" and 100 NMF replicates were used. A range of signature numbers were explored, ranging from 1-10 for the per-individual analysis, and 1-3 for the per-family analysis (above 3 there were too many signatures for the number of input samples when individuals were grouped per family). The optimal solution that maximizes stability while minimizing cosine similarity was chosen by the software: for each analysis (per-individual and per-family), one signature was chosen as the optimal solution.

The cosine similarity between the optimal reconstructed mutation spectra and the empirical data ranged from 0.563--0.821 in the per-individual analysis, from 0.803-0.882 in the per-family analysis.

The optimal single signature in each analysis was deconvoluted by SigProfilerExtractor into contributions from known COSMIC (Catalogue of Somatic Mutations in Cancer) signatures. In each case, the extracted signature was deconvoluted into signatures SBS1 and SBS5, two clock-like signatures that generally make up the bulk of mutations in both germline and somatic data. No contributions of SBS18 or SBS36, somatic mutational signatures associated with defective *MUTYH*, were detected.
